# Combined robotic inguinal and iliac‐obturator lymphadenectomy for stage III skin cancers: Surgical technique and preliminary results

**DOI:** 10.1002/rcs.2391

**Published:** 2022-03-17

**Authors:** Elisa Francone, Simona Reina, Francesco Spagnolo, Lorenzo Di Maira, Ferdinando Cafiero, Nicola Solari

**Affiliations:** ^1^ General Surgery 1 Unit IRCCS Ospedale Policlinico San Martino Genova Italy; ^2^ Medical Oncology 2 Unit IRCCS Ospedale Policlinico San Martino Genova Italy

**Keywords:** iliac dissection, Indocyanine‐green fluorescence, inguinal dissection, melanoma, Merkel cell carcinoma, robotic lymphadenectomy, robotic lymph node dissection

## Abstract

**Background:**

Ilio‐inguinal lymphadenectomy for stage III melanoma and skin cancers still represents the best therapeutic option for a subset of patients, although the incidence of post‐operative complications is dramatically high. Only a paucity of papers on robotic approach have been published, reporting experiences on isolated pelvic or inguinal lymphadenectomy, and no series on combined dissections have been described yet. We present the preliminary results achieved with combined robotic approach, with special emphasis on lymph nodal mapping, dissection technique and postoperative complications linked with the lymphatic system.

**Methods:**

Between September 2019 and September 2021, 10 patients were submitted to robotic inguinal and iliac‐obturator lymphadenectomy.

**Results:**

Post‐operative course was characterised by early mobilisation and minimal post‐operative pain. Only one lymphoedema occurred and lymph nodal harvesting was more than satisfactory.

**Conclusions:**

Robotic surgery provides meticulous lymph nodal dissections, with promising functional and oncologic outcomes. Further series are advocated to confirm these preliminary results.

## 1 INTRODUCTION

According to the National Comprehensive Cancer Network (NCCN) guidelines on cutaneous melanoma, complete lymph node dissection (CLND) is the treatment of choice in patients presenting with clinically/radiologically involved lymph nodes (LNs), without radiologic evidence of distant metastases, providing a 5‐year survival between 30% and 50%.^1^ NCCN guidelines on Merkel cell carcinoma (MCC) suggest instead the aggressiveness commensuration of nodal dissection with the extent of nodal disease, considering that patients with clinically positive nodal disease have pathologically involved LNs in 60%–100% of cases when dissected.^2^ However, despite the prognostic and therapeutic value of CLND, the procedure is burdened with high morbidity rates, ranging from 19% to 77%.^3^ In particular, ilio‐inguinal dissection is historically associated with wound complications, lymphatic fistulas, seroma/lymphocele formation and chronic lymphoedema, with prolonged in‐hospital stay and severe impairment in quality of life.^3^, ^4^, ^5^ In order to overcome these criticisms, novel minimally invasive alternatives to the conventional open approach have been introduced, including videoscopic inguinal and iliac‐obturator lymphadenectomy (VIIOL), alone or in combination.^4^, ^5^, ^6^, ^7^ As discussed in our single‐institution experience, a combined VIIOL minimises surgical morbidity and accelerates recovery of daily activities, maintaining appropriate oncologic outcomes.^6^

With the effort to improve stage III melanoma and more generally stage III skin cancers oncological care, a paucity of papers on robotic approach have been recently published, but the reported experiences only concern isolated pelvic^8^, ^9^, ^10^, ^11^ or inguinal dissections.^12^ As far as we know, no series on combined robotic inguinal and iliac‐obturator lymphadenectomy (RIIOL) have been described yet.

We present the surgical technique and related outcomes of RIIOL for stage III skin cancers, deepening current and perspective applications of this innovative technique, with special emphasis on lymph nodal dissection technique enhanced by high‐resolution images magnification and Indocyanine‐green (ICG) real‐time fluorescent technology application, weapons to accurately evaluate lymphatic mapping and eventual lymphatic leak, possibly reducing postoperative complications linked with the lymphatic system.

## 2 PATIENTS AND METHODS

Between 1^th^ September 2019 and 30^th^ September 2021, patients presenting with cutaneous melanoma or MCC and either clinical or radiologically confirmed positive inguinal or iliac LNs in the absence of distant metastases have been submitted to RIIOL at the IRCCS Ospedale Policlinico San Martino, Genova, Italy. Indications for surgery were discussed by a multidisciplinary team. Absolute contraindications for the robotic approach were severe cardiac or respiratory failure. Every patient signed an informed consent form. Baseline characteristics including age, sex, body mass index (BMI), comorbidities, primary tumour location and histology have been recorded. Intra‐ and postoperative outcomes have also been collected, including type of surgery and surgical technique adopted, operative duration, hospital stay, length of drain placement and volumes of drainages and complication rate according to Clavien‐Dindo Classification,^13^ particularly focussing on lymphoedema occurrence. Limb circumference measurements to assess postoperative lymphoedema were performed for both legs preoperatively, and then patients were followed up at 1, 3 and 6 months after surgery. According to our Institutional protocol for lymphoedema recognition, measurements were done at the superior border of the patella, 10 cm above and below the superior border of the patella, at the ankle and at the dorsum of the foot.^14^

The study was approved by the Institutional Ethical Review Board of our Institution (IRB approval number: CER Liguria 617/2021).

## 3 OPERATIVE TECHNIQUE

The night before surgery every patient receives subcutaneous low molecular weight heparin (dosage according to the weight), that will be taken for another 30 days. At the time of surgery, patients wear compression stockings, kept until a normal deambulation has been restored. Two grams of prophylactic ev Cefazolin are administrated and urinary catheter is placed.

Combined pelvic and inguinal robotic technique includes two surgical steps: the abdominal time for iliac‐obturator LNs dissection, and the inguinal time for inguinal LNs dissection. The procedure is borrowed from the laparoscopic approach we already described.^6^ Briefly, under general anaesthesia, the patient is placed in the supine position with the pelvis slightly extended on a split‐leg table tilted 30° up on the side to treat, in head‐down position.

**FIGURE 1:**
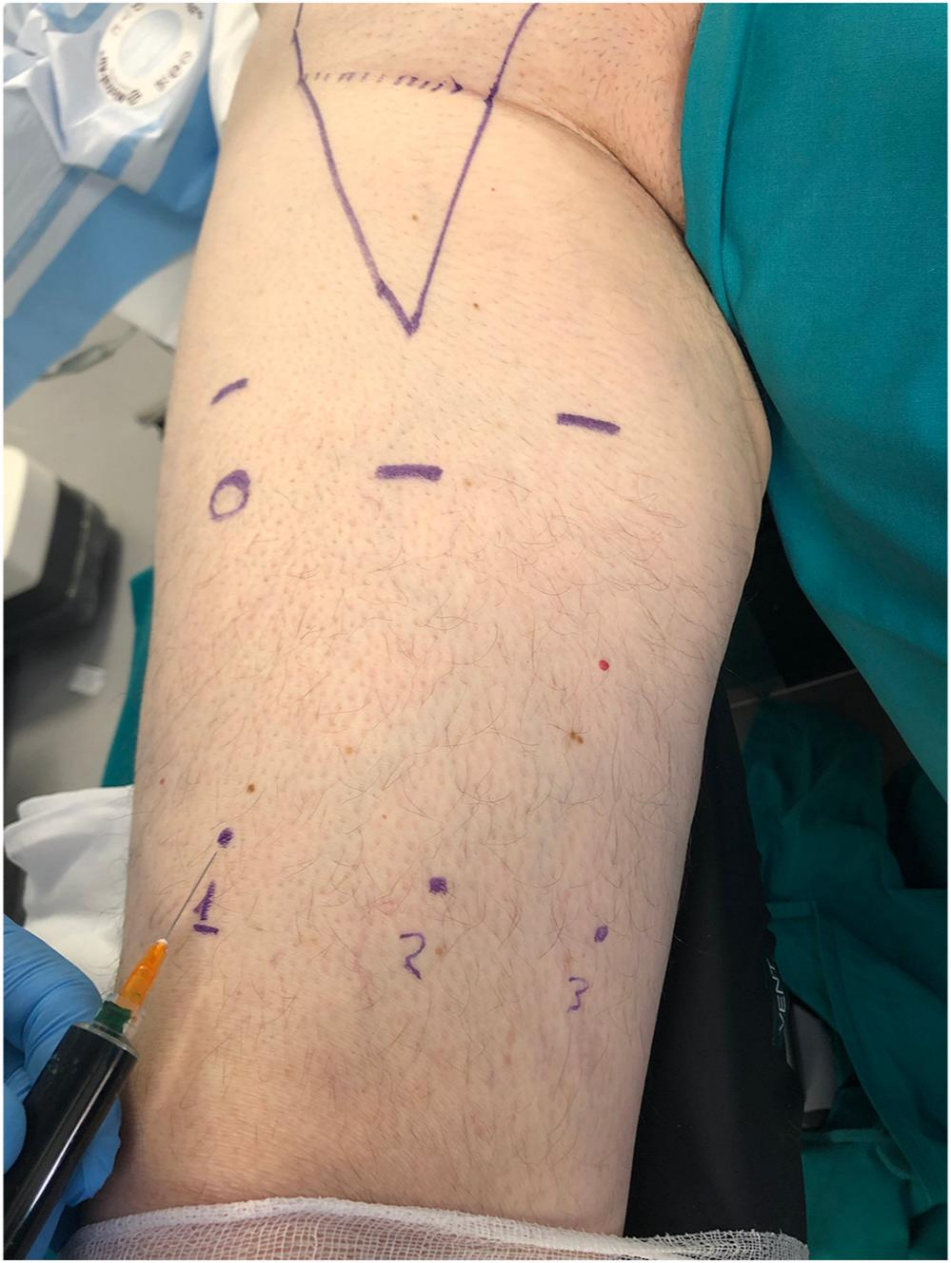
Three points of injections of 1 ml of Indocyanine‐green (ICG) each, are performed preoperatively on a transverse line running from the medial aspect to the anterolateral margin of the lower third thigh, including dermis, subcutaneous tissue and muscular fascia

For the study of lymphatic mapping, three injections of 1 ml of ICG each are performed preoperatively on a transverse line running from the medial aspect to the anterolateral margin of the lower third thigh, including dermis, subcutaneous tissue and muscular fascia, in order to distributing the solution along the lymphatic afferent pathways leading to the limb root (Figure 1).

### 3.1 Pelvic step

**FIGURE 2:**
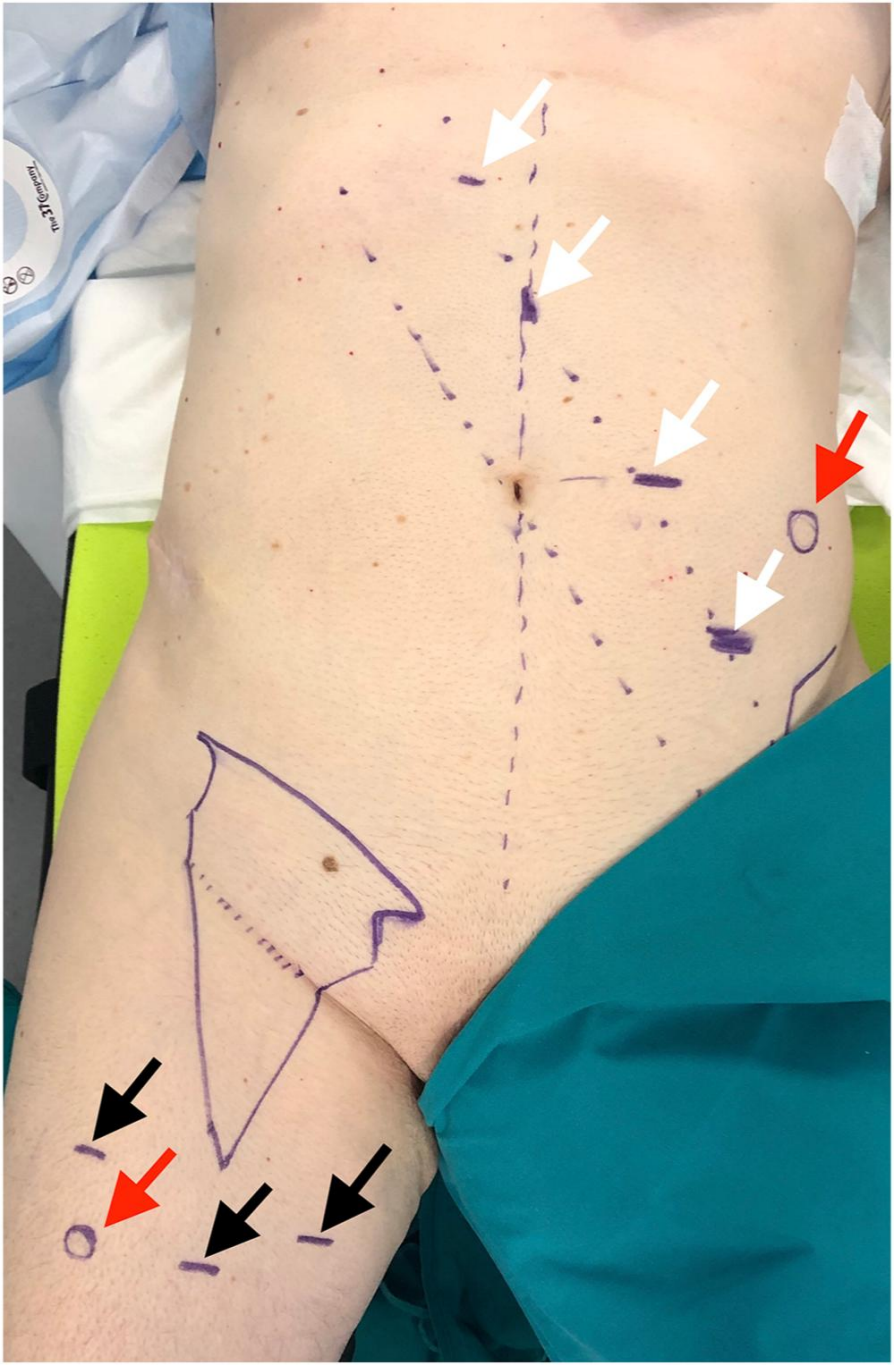
Ports positioning. White arrows indicate robotic ports position for pelvic step. Black arrows indicate robotic ports position for inguinal step. Red arrows indicate service trocars position

The pelvic step is performed via four plus one service ports. The procedure starts inserting an 8 mm peri‐umbilical port, contralateral to the side to treat. Pneumoperitoneum is established at a mean pressure of 8 mmHg; three additional 8 mm ports are placed under laparoscopic vision, along an ideal line passing through the contralateral anterosuperior iliac spine and the contralateral hypochondrium, spaced about 8 cm from each other. Finally, a service 12 mm port hosting the AIRSEAL Intelligent Flow System^®^ (ConMed; Utica, New York, USA) is placed in the ipsilateral iliac fossa (Figure 2). The robotic cart approaches the operative table from the ipsilateral side, serving for both pelvic and inguinal times. In the docking phase the optics are pointed at the inner inguinal ring; robotic arms are then connected to the trocars and instruments (dissector, monopolar and cadiere forceps) are introduced.

Dissection is conducted with monopolar forceps. The lateral border of the dissection is developed along the genito‐femoral nerve by dissecting the fibro‐areolar tissue and exposing the ilio‐psoas muscle. The lymphatic tissue packet is lifted off the surface of the ilio‐psoas muscle and swept medially. External iliac vessels are circumferentially skeletonised using monopolar dissector, with common iliac arteries bifurcation representing the proximal border. Hypogastric artery is carefully mobilised in order to avoid injuries to the internal iliac vein. The released packet is rolled medially on the back side of the mobilised external iliac artery and vein, delivering it into the pelvis. Dissection along the medial aspect of the packet allows the identification of the obturator nerve. Distally, lymphatic vessels caudal to the Cloquet's node are clipped and transected. The entire specimen is immediately placed in an endo‐catch to avoid any spillage of cancer cells.

At the end of the dissection the modality of vision is switched to near‐infrared light, in order to identify any residual node and any lymphatic leak, that can be immediately managed by clipping the leaking lymphatic vessels. The procedure is completed by peritoneal repair with a running suture. A 15‐G fluted drain is left in site.

### 3.2 Inguinal step

For the inguinal step a three plus one service ports technique is used, with the first 10 mm port placed 3 cm distally to the apex of the femoral triangle. The skin is incised and sharply dissected down through Camper and Scarpa's fascias. The working‐space is then developed by blunt‐finger dissection, extending out 5 cm on each side from the incision.

Two more 8 mm robotic ports are positioned 3 cm outside of the medial and lateral boundaries of the previously delineated femoral triangle while a service 12 mm port is positioned under the lateral port (Figure 2).

**FIGURE 3:**
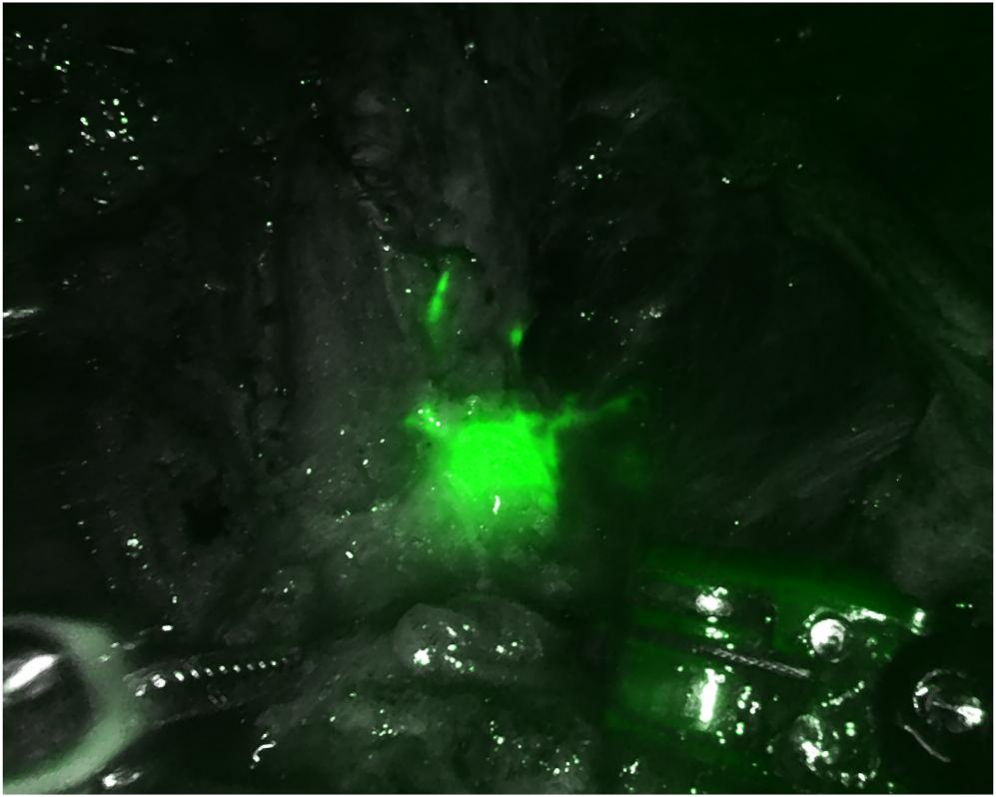
Operative field after inguinal lymphadenectomy: Indocyanine‐green (ICG) real‐time fluorescent technology allows identifying residual lymph nodes (LNs)

**FIGURE 4:**
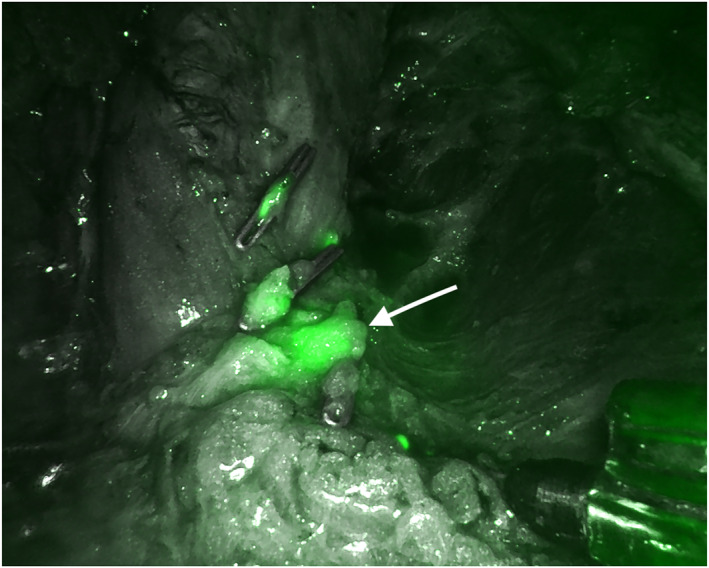
Operative field after inguinal lymphadenectomy: Indocyanine‐green (ICG) real‐time fluorescent technology allows identifying lymphatic leak (arrow)

**FIGURE 5:**
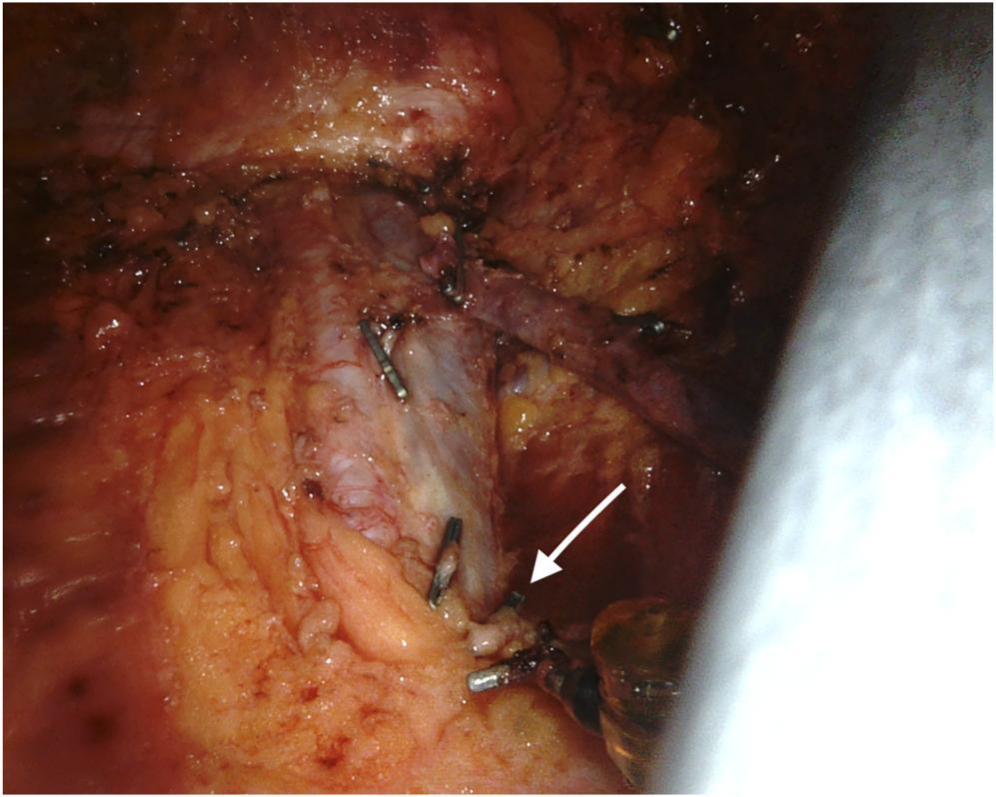
Operative field after inguinal lymphadenectomy: A clip has been positioned to secure the lymphatic vessel responsible for the leak (arrow)

The new docking phase is carried out, pointing the optics cranially at 12 o'clock. The working‐space is insufflated up to 10 mmHg. To complete the definition of the anterior space between the fibro‐adipose node‐containing packet and the superficial tissue, a monopolar dissector is used. Pressure is then reduced to 6 mmHg to prevent end‐tidal CO2 elevation. Anatomical boundaries include: the sartorious muscle, laterally; the abductor muscle, medially; the external oblique fascia and inguinal ligament, proximally; and the apex of femoral triangle, distally. The saphenous vein is readily identified within the apex of the femoral triangle, if indicated then closed with clips and divided with monopolar dissector. Careful dissection within the femoral triangle enables the identification of the femoral artery pulse as well as the medial femoral vein. An endoscopic linear cutting stapler with a vascular load or vascular clips are used to transect the vein at the sapheno‐femoral junction. The nodal packet is withdrawn with endo‐bag through the apical port. At the end of the dissection, near infra‐red light is turned on to verify the accuracy of lymphadenectomy and eventual lymphatic leaks (Figures 3, 4, 5); if residual pathological LNs are found, once the internal circumflex vessels, internal pudendal vessels and, if necessary, inferior deep epigastric vessels are isolated and transected, those nodes are easily removed avoiding the transection of the inguinal ligament. The procedure is completed by placing an 18‐G fluted drain through the medial port site.

Postoperative pain is managed with paracetamol administered when necessary. No oral antibiotics are routinely prescribed after surgery. Patients are given a regular diet and encouraged to walk on the first post‐operative day.

## 4 RESULTS

Over a period of 25 months, 10 patients (7 males, 3 females) with a mean age of 63.8 years (range 21–91 years), and a mean BMI of 26.8 kg/m^2^ (range 22.04–31.25 kg/m^2^) met the inclusion criteria for participation. In particular 7 patients have been submitted to combined RIIOL, while 3 patients underwent lone robotic inguinal lymphadenectomy (RIL).

Histotype of the primary tumour was melanoma of the trunk in 4 cases, melanoma of the lower limb in 4 cases, and MCC of the lower limb in 2 cases.

Surgical procedures were well tolerated in all cases, and no conversion to laparoscopic nor open approach occurred. Mean operative time was 330 min (range 280–405 min) for RIIOL and 211 min (range 180–240 min) for RIL. Overall, blood loss was insignificant for both procedures and no blood transfusions were required.

Post‐operative course was characterised by prompt removal of the bladder catheter at the end of surgery, early mobilisation on post‐operative day zero, and minimal post‐operative pain, requiring just in 3 cases the administration of minor analgesics (paracetamol 1000 mg).

Mean in‐hospital stay was 2 days (range 1–3 days) for both procedures.

Drains were usually left in situ until the volume of the drainage was inferior to 50 ml per day, meaning for an average of 23 days (range 21–28 days).

Histopathological evaluations revealed an average of 19 LNs (range 9–27 LNs) harvested for RIIOL, and an average of 6,3 LNs (range 5–9 LNs) for RIL.

Patients' characteristics data are summarised in Table 1.

**TABLE 1:** Patient's characteristics

Patient number	Sex	Age	BMI	DM	Smoking	Primary tumour histology	Primary tumour location	Surgical technique	Number of LNs/case	Number of LNs/case iliac‐obturator	Number of LNs/case inguinal	Saphena ‐sparing	Use of ICG	ICG identified residual nodes
1	M	57	24.49	NO	NO	MM	Trunk	RIIOL	15	6	9	YES	NO	n/a
2	M	74	27.68	NO	NO	MM	Trunk	RIL	5	N/A	5	YES	YES	0
3	M	72	27.78	NO	NO	MCC	Lower limb	RIIOL	15	8	7	NO	NO	n/a
4	F	51	22.04	NO	NO	MM	Lower limb	RIIOL	20	11	9	NO	YES	1
5	M	72	29.07	NO	NO	MM	Lower limb	RIL	5	N/A	5	NO	YES	1
6	M	74	26.12	NO	NO	MM	Trunk	RIIOL	9	4	5	YES	YES	0
7	F	54	31.25	NO	NO	MCC	Lower limb	RIIOL	27	20	7	NO	YES	0
8	F	91	27.78	NO	NO	MM	Trunk	RIL	9	N/A	9	YES	YES	0
9	M	72	29.38	NO	NO	MM	Lower limb	RIIOL	26	7	19	NO	YES	0
10	M	21	22.86	NO	NO	MM	Lower limb	RIIOL	21	8	13	NO	YES	2

Abbreviations: BMI, body mass index (kg/m^2^); DM, diabetes mellitus; F, female; ICG, Indocyanine‐green; LNs, lymph nodes; M, male; MCC, Merkel cell carcinoma; MM, malignant melanoma; N/A, not applicable; RIIOL, robotic inguinal and iliac‐obturator lymphadenectomy; RIL, robotic inguinal lymphadenectomy.

Only one patient submitted to RIIOL developed a Grade II complication according to Clavien‐Dindo Classification,^13^ consisting in postoperative wound infection, managed with oral antibiotics. Three patients developed Grade I complication consisting in postoperative seroma managed conservatively. With a mean follow‐up of 11 months, 1 patient presenting with melanoma of the lower limb submitted to RIIOL developed lymphoedema. No port‐side recurrence occurred.

## 5 DISCUSSION

Despite indications for ilio‐inguinal lymphadenectomy in stage III melanoma and skin cancers are steadily resized, there's still a subset of patients for whom CLND represents the best therapeutic option.^1^, ^10^ Unfortunately, the incidence of CLND morbidity still remains dramatically high, impairing patients' quality of life also in the long term.^1^, ^3^, ^6^ But in an era of continuous improvements in cancer management paradigms, surgical practice should guarantee oncological standards, minimising at the same time physical traumas and complications.

Over the past few years, several groups ‐ including ourselves ‐ reported their experience with miniinvasive approaches to skin cancers, mostly videoscopic, showing optimal functional and cancer‐related outcomes.^4^, ^5^, ^6^, ^7^ In fact, avoiding large incisions reduces the chance of wound‐related morbidity, without impairing accurate dissections.^4^, ^5^, ^6^ Thanks to the rapid expansion of robotic surgery, preliminary enthusiastic results have been recently reported for isolated pelvic or inguinal basin.^8^, ^9^, ^10^, ^11^, ^12^ Robotic surgery can indeed be particularly effective in narrow fields, fostering the surgeon in performing subtle movements by avoiding the encumbrance of laparoscopic instruments, thus being particularly indicated for pelvic lymphadenectomy (especially when dealing with male and obese patients) but even more for inguinal dissection, where the working‐space is limited and superficial. Actually, there is controversy on the specific number of LNs to be harvested to define optimal CLND in melanoma patients, with NCCN practice guidelines recommending to fully describe the anatomic boundaries of the field of dissection.^1^ Introducing robotic approach, we maintained optimal lymph nodal dissection average, with only one patient experiencing lymphoedema. As a matter of fact, ICG real‐time fluorescent technology allows accurate evaluation of lymphatic mapping and early recognition of lymphatic leak, possibly reducing postoperative complications linked with the lymphatic system, lymphoedema included.^15^ Furthermore, thanks to the sharpness of high‐resolution images magnification of robotic equipment, a meticulous anatomical dissection can be pursued, facilitating complete LNs removal and enabling saphena‐preserving procedures when indicated (i.e. in the case of melanoma of trunk; Figure 5). The effect of preserving the saphenous vein is a matter of debate, since both a reduction or no advantages in the rate of chronic lymphoedema occurrence have been reported.^16^, ^17^ On the contrary, oncological implications have only been investigated in a rudimentary way, and the option to resect or spare saphenous vein still remains facultative.^16^ According to our Institutional trend, no conservative procedures on the great saphenous vein in patients with lower extremities malignancy have been attempted,^14^ and although the lone lymphoedema occurred in this series was in a lower limb melanoma patient with resected saphenous vein, the small number of patients of this pilot experience doesn't allow to draw any conclusion on the topic.

In the presented series, mean operative time for combined RIIOL is 330 min, a moderately longer duration if compared with our laparoscopic experience of 302 min.^6^ However double robotic docking is time‐consuming di per se, and since this cohort comprehends patients operated during our learning curve period, a shortening in surgery duration is expected. Indeed, as we previously reported in our experience with combined VIIOL,^6^ even with the robotic approach we assumed that a combined procedure can emerge the advantages of the two separate surgeries, pelvic and inguinal.

Length of hospital stay is 2 days, comparable with videoscopic procedure.^6^ The most relevant difference from videoscopy has been a further reduction of post‐operative pain, managed with paracetamol only, administered when necessary by the first post‐operative day.

Our preliminary experience with combined RIIOL is still limited and the follow‐up restricted, therefore we can't still draw firm conclusions, but as for other surgical procedures, robotic approach seems to be a promising weapon in managing stage III skin cancers, allowing meticulous lymph nodal dissections, with promising functional and oncologic results.

Further investigations on larger series are advocated to confirm these preliminary findings.

## CONFLICT OF INTEREST

The Authors have no conflict of interest to declare.

## Data Availability

The data supporting the findings of this study are available from the corresponding author upon reasonable request.
